# Supplementary material for: Antioxidant Effects of Anthocyanin-Rich Riceberry™ Rice Flour Prepared Using Dielectric Barrier Discharge Plasma Technology on Iron-Induced Oxidative Stress in Mice
Source: Molecules. 2021 Aug 17;26(16):4978. doi: 10.3390/molecules26164978 (PMC8399969; doi:10.3390/molecules26164978)
Supplement: Supplementary file 1 [file molecules-26-04978-s001.zip › molecules-1320222-supplementary.pdf]

Supplementary Materials

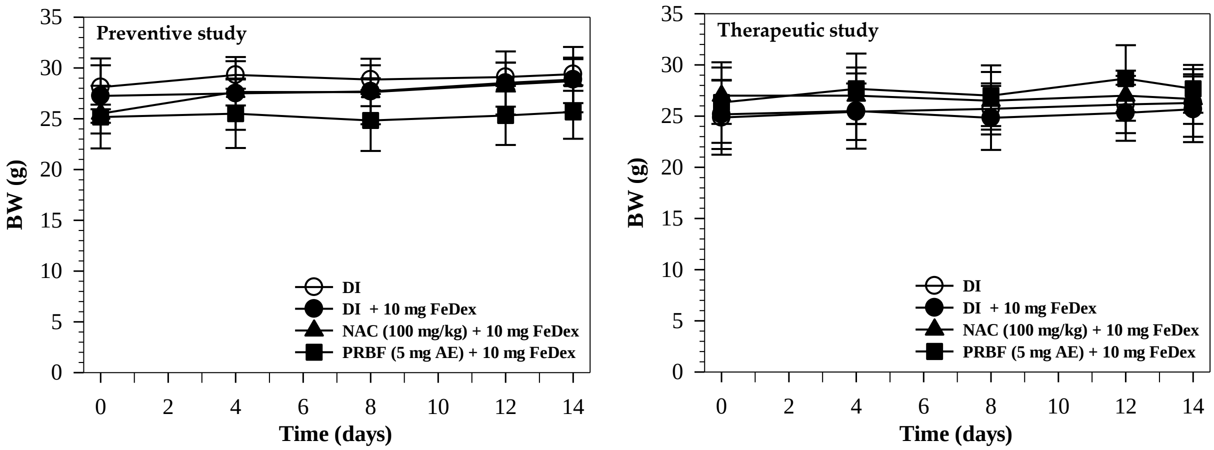

Figure S1. BW changes of FeDex-loaded mice treated with DI, NAC and PRBF. In the protective study, mice were treated with DI, NAC (100 mg/kg) and PRBF (5 mg AE/kg).This was followed by a single ip injection with 10 mg FeDex after the last oral dose was administered on day 14, at which point BW was recorded. In the therapeutic study, mice were ip injected with 10 mg FeDex together with the oral administration of DI, NAC (100 mg/kg) and PRBF (5 mg AE/kg) for 14 d and BW was recorded. Data are expressed as mean + SD values (n = 6). Abbreviations: AE = anthocyanin equivalent, BW = body weight, PRBF = plasma-treated Riceberry rice flour, DI = deionized water, FeDex = iron dextran, NAC = N-acetylcystein, SD = standard deviation.

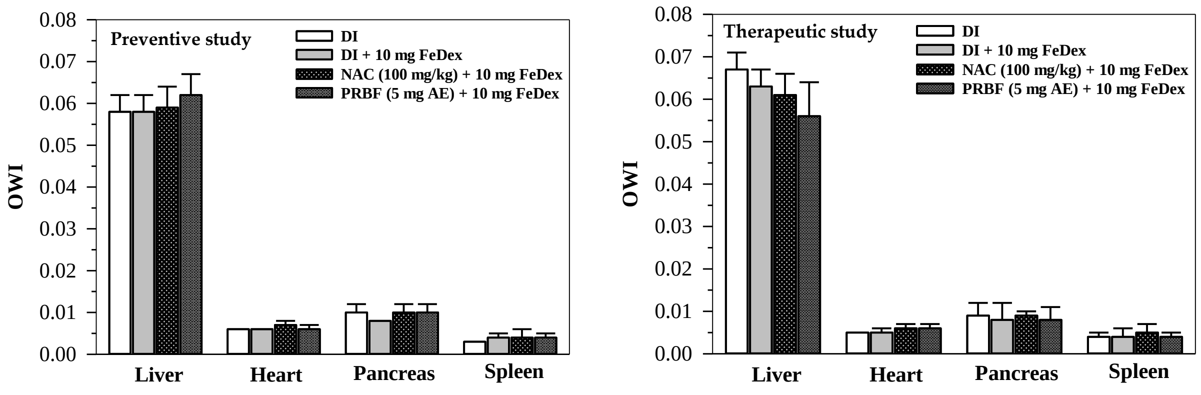

Figure S2. OWI values of the liver, heart, pancreas and spleen taken from FeDex-loaded mice that had been treated with DI, NAC and PRBF. In the protective study, mice were treated with DI, NAC (100 mg/kg) and PRBF (5 mg AE/kg) for 14 d. This was followed by a single ip injection with 10 mg FeDex after the last oral dose was administered on days 14. Mice were then sacrificed; their internal organs were detected and their OWI values were determined. In the therapeutic study, mice were ip injected with 10 mg FeDex together with DI, NAC (10 mg/kg) and PRBF (5 mg AE/kg) for 14 d. Mice were then sacrificed, their internal organs were dissected and their organ weight indices (OWI) were determined. Data are expressed as mean+SD values (n = 61). Abbreviations: AE = anthocyanin equivalent, PRBF = plasma-treated Riceberry rice flour, DI = deionized water, FeDex = iron dextran, NAC = N-acetylcystein, OWI = organ weight index, SD = standard deviation.
